# The Effect of High Hydrostatic Pressure (HHP) Induction Parameters on the Formation and Properties of Inulin–Soy Protein Hydrogels

**DOI:** 10.3390/gels10090570

**Published:** 2024-08-31

**Authors:** Anna Florowska, Tomasz Florowski, Patrycja Goździk, Adonis Hilal, Hanna Florowska, Emilia Janiszewska-Turak

**Affiliations:** 1 Department of Food Technology and Assessment, Institute of Food Sciences, Warsaw University of Life Sciences-SGGW, 159c Nowoursynowska Street, 02-787 Warsaw, Poland; tomasz_florowski@sggw.edu.pl (T.F.); adonis_hilal@sggw.edu.pl (A.H.); 2 Department of Food Safety and Chemical Analysis, Prof. Waclaw Dąbrowski Institute of Agricultural and Food Biotechnology-State Research Institute, 36 Rakowiecka Street, 02-532 Warsaw, Poland; hanna.florowska@ibprs.pl; 3 Department of Food Engineering and Process Management, Institute of Food Sciences, Warsaw University of Life Sciences-SGGW, 159c Nowoursynowska Street, 02-787 Warsaw, Poland; emilia_janiszewska_turak@sggw.edu.pl

**Keywords:** inulin, soy protein, HHP, hydrogels

## Abstract

The aim of this study was to determine the effect of high hydrostatic pressure (HHP) induction parameters on the formation and properties of inulin–soy protein hydrogels. Solutions containing 20 g/100 g of inulin and 3 or 6 g/100 g of soy protein isolate (3 SPI; 6 SPI) were subjected to HHPs of 150, 300, or 500 MPa for 5, 10, or 20 min. The HHP parameters had no significant impact on the effectiveness of hydrogel formation. In most cases, the time of solution pressurization had no significant effect on the characteristics of hydrogels. However, increasing the induction pressure from 150 to 300 MPa resulted in hydrogels with different characteristics being obtained, e.g., more flattened microstructure; higher stability (only 3 SPI); higher yield stress, firmness, and adhesiveness; and lower spreadability. These changes were more noticeable in the hydrogels with lower protein content. An increase in the induction pressure (to 500 MPa) did not result in a significant strengthening of the hydrogel structure. However, in the case of 6 SPI hydrogels, induction with a pressure of 500 MPa had an unfavorable effect on their stability. The results indicate that HHP (300 MPa) can be used as an effective method for strengthening the structure of inulin–protein hydrogels.

## 1. Introduction

Interest in the development of new food materials based on hydrogel polymers can be observed in the latest literature data [1]. Hydrogels are defined as hydrophilic polymers with a three-dimensional network structure that have the ability to immobilize a large amount of water due to the presence of hydrophilic moieties [2]. The unique properties of hydrogels such as texture, flow properties, and swelling abilities make their usage flexible and versatile. This is why hydrogels are applied in many industries, from agricultural and food to biomedical and pharmaceutical [3]. Recent trends in their use include biomedical engineering to soft robotics, research in the field of stimuli-responsive drug-loaded hydrogels [4], and shape memory hydrogels (SMHs) created for biomedical applications [5,6]. The properties of hydrogels depend mostly on their composition. Most popularly used food hydrogels are based on proteins or on polysaccharides, but to develop the functionality of hydrogels they might also be created as protein–polysaccharide mixtures [7]. The protein–polysaccharide hydrogels most frequently described in the literature are based on starch or pectin with the addition of protein of plant or animal origin [8,9,10]. Producers creating functional foods are, however, also interested in using polysaccharides, which are classified as prebiotic fibers [11], and plant proteins, which are a source of essential amino acids [12].

One of the most frequently used prebiotic ingredients in food products is inulin. It belongs to the group of non-digestible linear fructans, which are found in over 36,000 species of plants growing around the world, and is the second most abundant carbohydrate in nature after starch [13]. On an industrial scale, in Europe, inulin is isolated mainly from chicory roots by aqueous extraction; the obtained extract is purified and spray-dried, creating the commercial product of white, odorless powder [14,15]. Inulin consists of fructosyl moieties linked by β-(2,1)—glycosidic bonds and might be linked at the non-reducing end to a terminal glucose residue by an α-(1,2) glycosidic bond (GFn). The hydroxyl groups present in the molecular structure of inulin can interact strongly with water, forming hydrogen and molecular bonds between inulin molecules, thus leading to a stable gel structure [16]. Inulin is classified as a prebiotic fiber, i.e., an indigestible substance that reaches the intestine in an unchanged form, where it undergoes complete fermentation and enables changes in both the composition and activity of the gastro-intestinal probiotic microflora, thus improving the host’s health [17].

In the food industry, inulin is an ingredient used as an additive to meat products, bakery products, breakfast cereals, extruded snacks, dairy products, and frozen desserts, increasing not only the nutritional value of the finished product, but also improving its organoleptic and textural characteristics. The texture-forming properties of inulin result from its ability to form three-dimensional particle gel networks from aqueous solutions in which a large amount of water is immobilized. The properties of inulin gels—including consistency, spreadability, the feeling of fullness, and a fatty mouthfeel—are very similar to fats. This is why inulin gels are used in many food applications as fat replacers [14,18]. Unfortunately, inulin gels are unstable; they easily liquify under the influence of applied force and are sensitive to changes in temperature and pH [19,20].

To improve the properties of inulin hydrogels, a binary gel can be created by combining inulin with protein [21]. One of the most common proteins used continuously in the food industry is soy protein. Soy proteins are commonly used in food production because they are a cheaper alternative to animal proteins, have a high nutritional value (they contain all the amino acids essential for the human body), and have functional properties (emulsifying, water-binding, and texturing properties). Soy protein isolates consist almost entirely of two fractions of globular proteins that vary in sedimentation rate: 7S and 11S. They have gelling properties and are good fat substitutes. The resulting protein gels are formed by internal and intermolecular forces (between protein and water molecules), including disulfide, hydrogen, and ionic bonds, and the hydrophobic effect [22,23,24].

Few inulin–protein hydrogels have been described in the literature. It is known that inulin can help unfold myofibrillar molecules and form a three-dimensional network structure through conformational conversion and chemical force formation to improve surimi gel characteristics [16]. Inulin can also inhibit gluten aggregation, thereby reducing hydrophobic interactions and disulfide bonds, forming a soft inulin–gluten gel [25]. Adding proteins to aqueous inulin solutions significantly affects the structure of the obtained inulin gels. As the protein concentration increases from 1 to 6%, the structure of the hydrogels becomes smoother and more coherent, as well as less granular [11].

Apart from the type and concentration of protein and polysaccharide components, the properties of hydrogels are also influenced by the induction method of these gels. In the case of inulin and protein, the most well-known methods of gel induction are thermal induction or the use of shear forces [26]. However, the formation of the gel structure is also possible using high-pressure homogenization (HPH) [21,27] and high hydrostatic pressure (HHP) [21,28]. The use of HHP technology contributes to a change in the properties of either polysaccharide gels or protein gels, and may thus also have an effect on binary polysaccharide–protein hydrogels. Although this has not been the subject of many published studies, it could enable their use in the areas of the food industry, where it was not possible until now [21]. Therefore, the aim of this work was to investigate the impact of high hydrostatic pressure (HHP) treatment, using different pressure parameters (150, 300, 500 MPa) and treatment times (5, 10, 20 min) on the formation and properties of polysaccharide–protein hydrogels formed with inulin and soy protein, aiming to tailor these biomaterials for targeted applications. In the available literature, there is a lack of research in this area, and taking into account the dynamics of the development of functional ingredients and non-thermal processing used in the food industry, such research seems to be extremely important.

## 2. Results and Discussion

### 2.1. The Effect of High Hydrostatic Pressure Induction Parameters on the Formation and Stability of the Obtained Hydrogels

#### 2.1.1. Volumetric Gel Index (VGI)

All tested inulin–soy protein solutions formed a gel structure (100% VGI) when subjected to high pressure. Effective gelation induction was achieved by subjecting the solutions to the lowest pressure (i.e., 150 MPa) for the shortest time (5 min), and the use of higher pressures and longer operating times did not have an adverse effect on the gelation of the tested solutions. A similar effect was previously observed when solutions containing only inulin (15–25 g/100 g) were subjected to high pressures at 150 and 300 MPa [29].

#### 2.1.2. Diffusing-Wave Spectroscopy (DWS)

The influence of high hydrostatic pressure on the characteristics of inulin–soy protein hydrogels was investigated using diffusing-wave spectroscopy (DWS). This technique monitors the Brownian motion of particles, enabling the non-invasive determination of the Mean Square Displacement (MSD) of particles across a range of frequencies as a function of time [30].

The evolution of MSD curves over time illustrates changes in microrheological properties during the gelation process of aqueous systems. Initially, a straight line in the MSD curves signifies a fluid-like state, with characteristics resembling Newtonian fluids [31]. The system undergoes thickening and crosslinking as time progresses, evident in the decreasing MSD values. This reduction reflects the formation of a network structure within the solution, typically achieved through the binding and water-holding capabilities of certain polymers like proteins or inulin [32]. The non-straight course of the curves begins at the moment of transition from liquid to sol, obtaining a “plateau” at the end of gelation that is typical of viscoelastic bodies in the middle decorrelation time [33].

**Figure 1:**
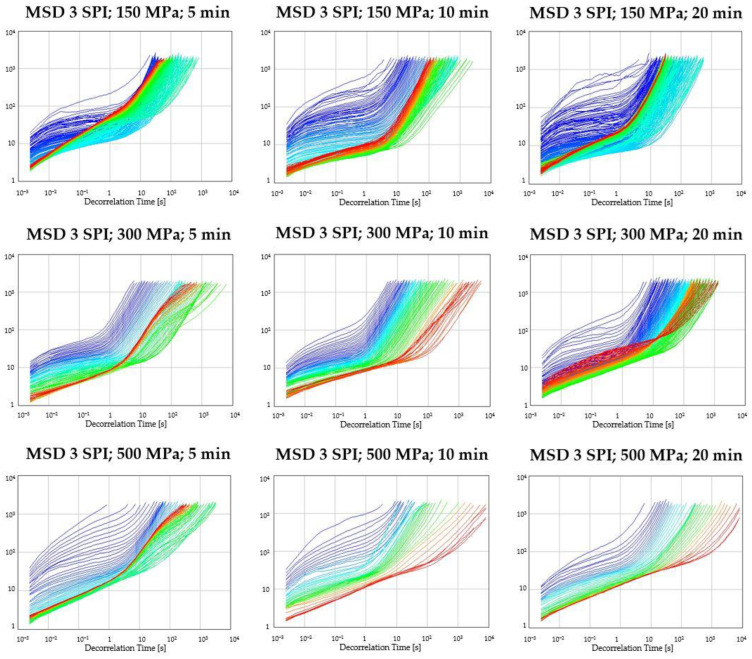
MSD (Mean Square Displacement) curves during gelation of aqueous inulin–protein solutions containing 3 g/100 g of soy protein isolate (24 h at 20 °C) after HHP treatment.

Figure 1 shows the MSD curves for the inulin–soy protein solutions exposed to selected pressures (150, 300, and 500 MPa) for specific times (5, 10, and 20 min), recorded for 24 h from sample preparation. These profiles do not include a complete transition from the liquid phase to the sol (within a short decorrelation time) because partial thickening of the solutions had already occurred at the stage of their preparation. After approximately 12 h, a significant increase in MSD was observed (regression of the curves), which is characteristic of the phenomenon of system destabilization—the release of water molecules (syneresis).

Analyzing the influence of the applied hydrostatic pressure and the duration of treatment on hydrogels containing 3 g/100 g of soy protein isolate, it can be concluded that increasing the pressure had a beneficial effect on the stability of the tested inulin–soy protein gels. However, only in the case of samples treated at 500 MPa for 10 or 20 min, no syneresis occurred (Figure 1). In addition, the MSD curves for these gels were the most uniform.

**Figure 2:**
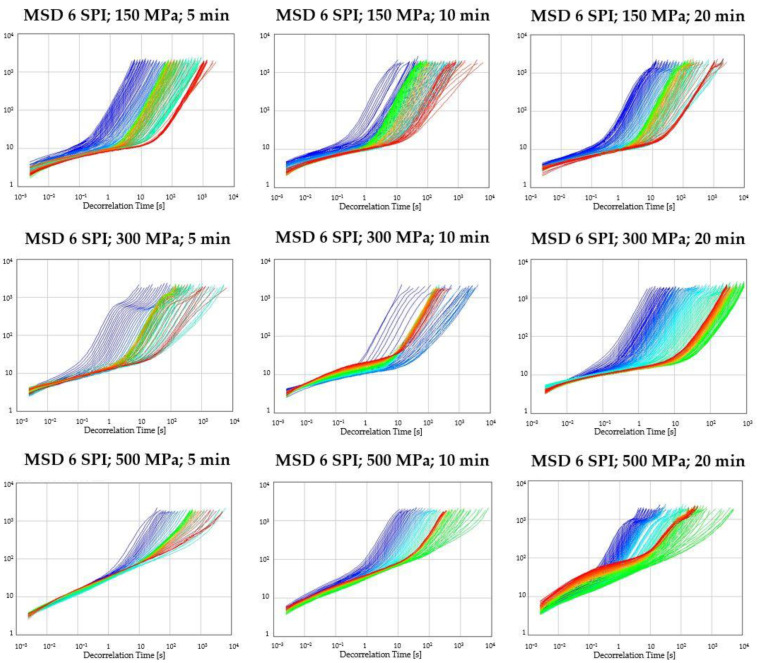
MSD (Mean Square Displacement) curves during gelation of aqueous inulin–protein solutions containing 6 g/100 g of soy protein isolate (24 h at 20 °C) after HHP treatment.

Studies have also shown that solutions containing higher SPI concentrations (6 g/100 g) exhibit narrower Mean Square Displacement (MSD) profiles compared to those with lower concentrations (3 g/100 g) during gelation (Figure 2). This phenomenon is attributed to an enhanced water binding capacity facilitated by the increased biopolymer content. In a model system employing whey proteins (4, 6, 8% *w*/*v*) and inulin (1–5%), Guo et al. [34] demonstrated that proteins acted as the dominant gelling agent. In contrast, inulin primarily influenced the elasticity of the formed gels. Additionally, Kim et al. [35] explain the influence of moderate pressures (100–300 MPa) as responsible for dissociating protein oligomers and aggregates, whereas relatively higher hydrostatic pressures (>300 MPa) are, according to the authors, for the denaturation of proteins. Pressure-induced protein unfolding is mainly caused by the elimination of water-excluded interior cavities and the hydration of hydrophobic surfaces, which can also be used for creating new connections with other polymers.

**Figure 3:**
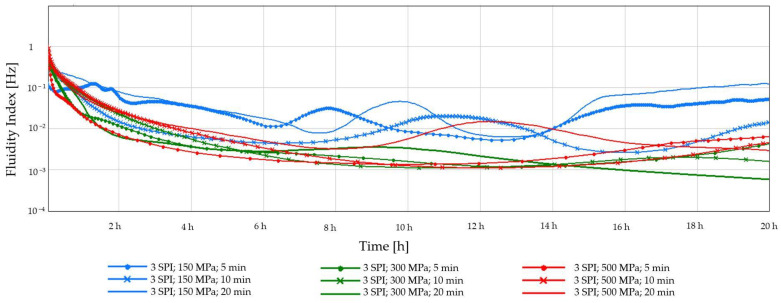
Changes in the fluidity index (FI) as a function of the gelation time of inulin–soy protein solutions (3 g/100 g) induced by HHP treatment with different pressure parameters (150, 300 and 500 MPa) and time (5, 10, and 20 min).

**Figure 4:**
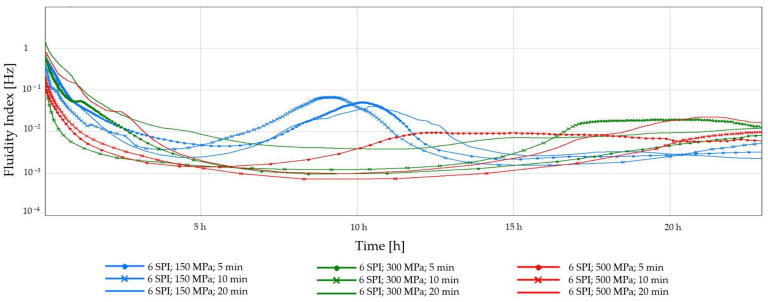
Changes in the fluidity index (FI) as a function of the gelation time of inulin–soy protein solutions (6 g/100 g) induced by HHP treatment with different pressure parameters (150, 300, and 500 MPa) and time (5, 10, and 20 min).

The early detection of gel destabilization is possible by monitoring changes in the fluidity index (FI), the low values of which (approx. 0.01 Hz) indicate the advantage of solid-state properties. As shown in Figure 3 and Figure 4, the preliminary FI decreased, indicating an initial aggregation of sol particles, leading to reduced fluidity. Maintaining the FI value at a constant level indicates viscoelastic equilibrium. The system’s physical destabilization is observed when this parameter’s value is increased. A sudden increase in the FI was observed in all samples obtained at the lowest pressure (150 MPa). A gradual increase in FI followed by a decrease may indicate slow gelation and forming a weaker, heterogeneous gel network [36]. If the FI is variable and unstable, it may also indicate that the structure of the gel is changing over time. In turn, the use of higher pressures (300 MPa and 500 MPa) and longer processing times (10 and 20 min) resulted in the formation of more compact and stable gel structures. Such relationships were observed regardless of the soy protein concentration used.

**Figure 5:**
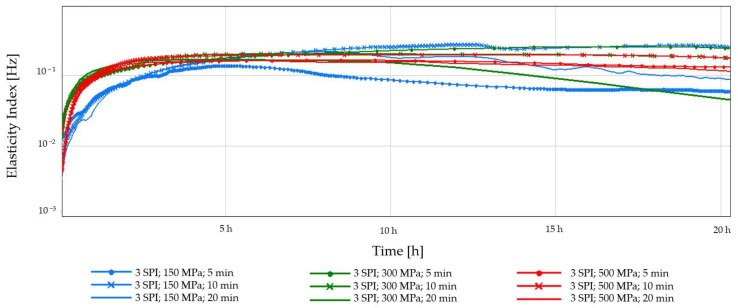
Changes in the elasticity index (EI) as a function of the gelation time of inulin–soy protein solutions (3 g/100 g) induced by HHP treatment with different pressure parameters (150, 300, and 500 MPa) and time (5, 10, and 20 min).

**Figure 6:**
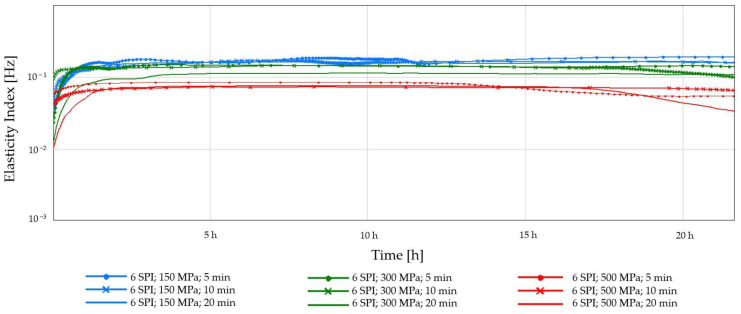
Changes in the elasticity index (EI) as a function of the gelation time of inulin–soy protein solutions (6 g/100 g) induced by HHP treatment with different pressure parameters (150, 300, and 500 MPa) and time (5, 10, and 20 min).

Figure 5 and Figure 6 show the elasticity index (EI) average values. Applying high hydrostatic pressure (HHP) to the samples induced conformational modifications in the biopolymers, facilitating the formation of an interconnected network through non-covalent interactions between inulin and protein molecules [27]. The greatest changes in time in EI were visible for samples subjected to the lowest pressure—150 MPa, especially for the lowest concentration of soy protein used (Figure 5). In the case of this concentration, there was a visible effect from either the applied pressure or the treatment duration. This may result from the fact that at lower pressure, the protein molecules may not unfold or aggregate as much, resulting in fewer crosslinks and a less rigid structure at a lower EI [37]. In the case of a higher protein concentration of 6%, the effect of the applied pressure on the EI is more clearly visible. The use of a higher pressure (300 MPa) resulted in the formation of less flexible, more compact gels, regardless of the exposure time. The results of this study are reflected in the higher stability and hardness of the obtained hydrogels. Applying HHP to protein solutions can significantly influence the formation and characteristics of the resulting gel networks. Studies have demonstrated that increasing pressure promotes novel intermolecular interactions between protein molecules, particularly hydrogen and disulfide bonds. These newly formed crosslinks serve to reinforce the gel matrix, leading to enhanced structural integrity and mechanical properties [38]. For the highest applied pressure, the effect of the duration of its treatment was observed. For the time of 20 min, a decrease in EI was observed. This may indicate that this high-pressure processing significantly impacts soy proteins. It is known that extensive HHP treatment can lead to wide protein unfolding, which can disrupt the ability of proteins to form strong interactions, leading to a weaker and less elastic gel network with a lower elasticity index [39].

**Figure 7:**
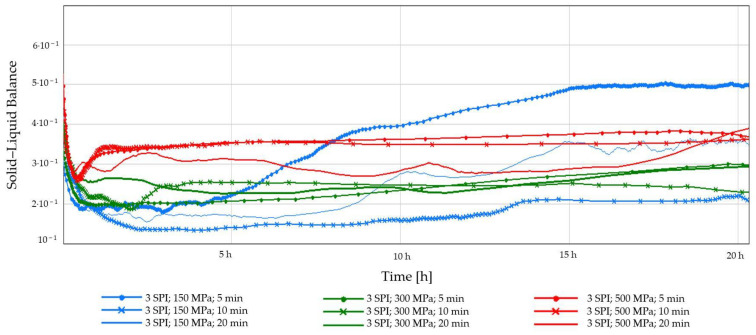
Changes in the Solid–Liquid Balance (SLB) as a function of the gelation time of inulin–soy protein solutions (3 g/100 g) induced by HHP treatment with different pressure parameters (150, 300, and 500 MPa) and time (5, 10, and 20 min).

**Figure 8:**
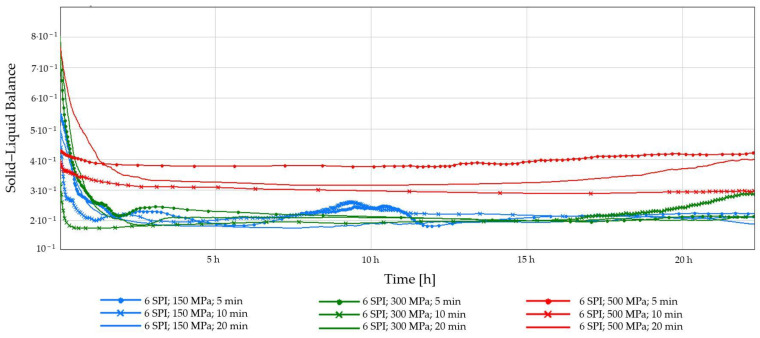
Changes in the Solid–Liquid Balance (SLB) as a function of the gelation time of inulin-soy protein solutions (6 g/100 g) induced by HHP treatment with different pressure parameters (150, 300, and 500 MPa) and time (5, 10, and 20 min).

The Solid–Liquid Balance (SLB) of the tested hydrogel samples is presented in Figure 7 and Figure 8. The SLB refers to the proportion of solid and liquid phases in a solution. For most of the tested samples, the SLB value remained constant, with the exception of gels obtained at the lowest pressure (150 MPa), regardless of the protein concentration. Hydrogels induced by a pressure of 150 MPa underwent changes during the measurement; their gel structure was weak and delicate, and their formation was accompanied by SLB changes over time. It was also observed that hydrogels obtained at the highest tested pressure—500 MPa—were characterized by the highest SLB values at both tested concentrations. The effect of high pressure can be explained by the fact that the application of HHP promotes the formation of a more solid-like gel network. This is due to the unfolding of protein molecules, exposing reactive groups that can participate in intermolecular interactions, like non-covalent interactions such as van der Waals, electrostatic, and hydrogen bonding. These interactions act as “crosslinks” between protein molecules, leading to a denser and more rigid network [40].

**Figure 9:**
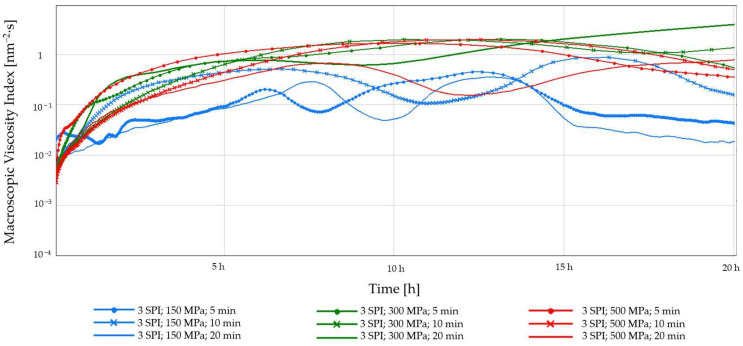
Changes in the Macroscopic Viscosity Index (MVI) as a function of the gelation time of inulin–soy protein solutions (3 g/100 g) induced by HHP treatment with different pressure parameters (150, 300, and 500 MPa) and time (5, 10, and 20 min).

**Figure 10:**
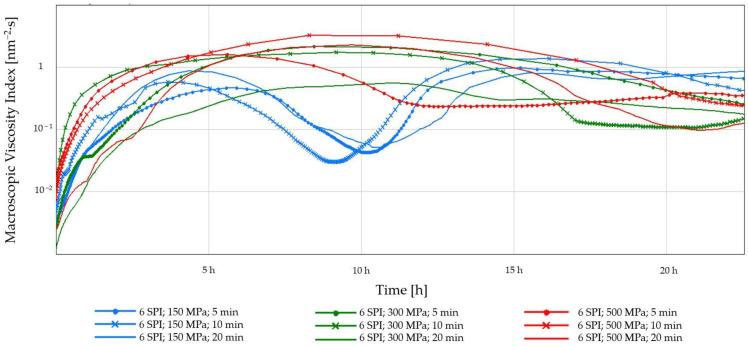
Changes in the Macroscopic Viscosity Index (MVI) as a function of the gelation time of inulin–soy protein solutions (6 g/100 g) induced by HHP treatment with different pressure parameters (150, 300, and 500 MPa) and time (5, 10, and 20 min).

The increase in Macroscopic Viscosity Index in the tested hydrogels, observed during the first 5 h of measurement (Figure 9 and Figure 10), indicates that the gel network formed and strengthened over time, and the dispersed particles in the suspension formed a three-dimensional network, trapping the liquid phase within its structure. Analyzing the trajectory of the MVI curves, it can be concluded that the gels obtained using the lowest tested pressure (150 MPa) also changed after 5 h, regardless of the initial protein concentration. This may be related to the continued crosslinking and densification, resulting in forming areas in the gel structure that allow the solvent to flow more easily [41].

#### 2.1.3. Centrifugal Stability Analysis Method (CSA)

**Figure 11:**
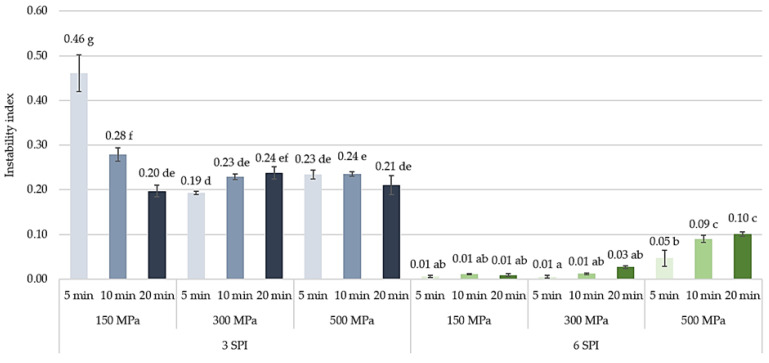
The effect of high hydrostatic pressure induction parameters on the stability of inulin–soy protein hydrogels (the average (n = 3) values marked with different letter symbols differ significantly (*p* < 0.05)).

Among the obtained inulin–soy protein hydrogels containing 3 g/100 g SPI, the hydrogel obtained after using the mildest high-pressure induction parameters, i.e., 150 MPa and 5 min, was characterized by the lowest stability. It was found that increasing the induction pressure from 150 to 300 MPa increased the stability of the resulting hydrogel (reducing the instability index from 0.46 to 0.19) (Figure 11). However, a further increase in the induction pressure, i.e., from 300 to 500 MPa, did not result in a further increase in the stability of the analyzed systems. The increase in the hydrogel stability obtained at the induction pressure of 150 MPa was also achieved by extending the high pressure exposure time from 5 to 10 or 20 min. It is also worth emphasizing that in the case of gels induced by the longest pressure time (i.e., 20 min), increasing the induction pressure from 150 to 300 or 500 MPa did not cause any significant changes in their stability. Another effect of HHP on the stability of inulin–protein hydrogels was observed in systems with a higher protein content. It was found that even the mildest high-pressure treatment of inulin–protein sols containing 6 g/100 g of SPI resulted in obtaining hydrogels with high stability (instability index 0.01). Increasing the induction pressure from 150 to 300 MPa and extending the induction time from 5 to 20 min had no significant effect on the stability of the formed hydrogels. It was found, however, that the use of higher induction parameters, i.e., a pressure of 500 MPa and an extension of the operation time, had an unfavorable effect on the stability of the obtained hydrogels. This effect was not observed in systems with lower protein content (i.e., 3 SPI).

**Figure 12:**
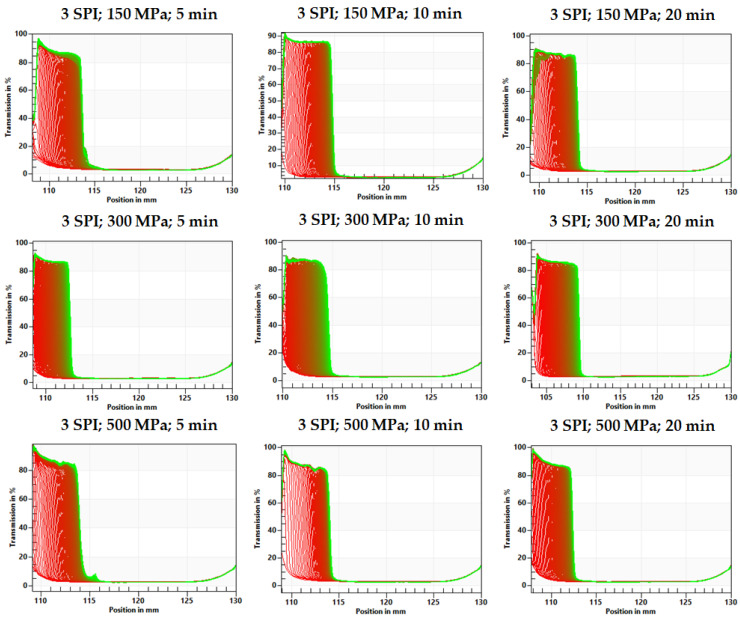
The effect of high hydrostatic pressure induction parameters on the inulin–soy protein (3 SPI) hydrogel transmission profiles presented enabling LUMiSizer^®^ analysis.

**Figure 13:**
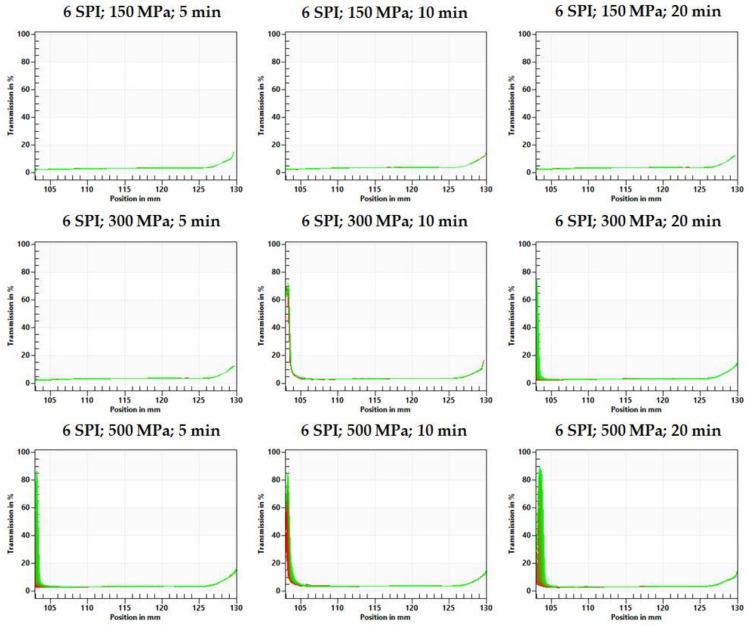
The effect of high hydrostatic pressure induction parameters on the inulin–soy protein (6 SPI) hydrogel transmission profiles presented enabling LUMiSizer^®^ analysis.

Based on the fingerprints, it can be seen that the gels obtained at lower protein concentrations (3 g/100 g) were already destabilized at the beginning of the analysis, and this destabilization progressed with the duration of the test (Figure 12 and Figure 13). The type of destabilization observed indicates the release of water in the upper part of the tested hydrogel samples. However, no differences in the resulting changes were observed between hydrogels obtained with different pressure process parameters.

Hydrogels obtained at higher protein concentrations (6 g/100 g) were characterized by much smaller changes in fingerprint profiles (Figure 13). Small changes were observed only at the highest applied pressure (500 MPa) and at 300 MPa for the longest time (20 min). The direction of changes also indicates the separation of a water layer on the sample surface. High hydrostatic pressure (HHP) induces extensive changes in the structure of proteins by disrupting electrostatic interactions and activating reactions of sulfhydryl–disulfide bond exchange [42]. Moreover, HHP treatments disrupt the electrostatic interactions stabilizing the quaternary and tertiary structure of proteins, leading to modifications in protein functional properties such as solubility, binding, and water absorption capacity, which are directly related to the stability of hydrogel systems [28]. According to the available literature, the applied pressure during HHP treatment affects the rate of these changes. For example, gels obtained from isolated hake myofibrils induced at 500 MPa were the most elastic and time-stable. They exhibited the highest level of connectivity [40], while for myofibrillar protein gel, the optimum gel-forming properties were achieved at a pressure of 200 MPa, and higher pressures caused a decrease in, among other factors, water-holding capacity [43].

### 2.2. The Effect of High Hydrostatic Pressure Induction Parameters on the Microstructure of the Obtained Hydrogels

**Figure 14:**
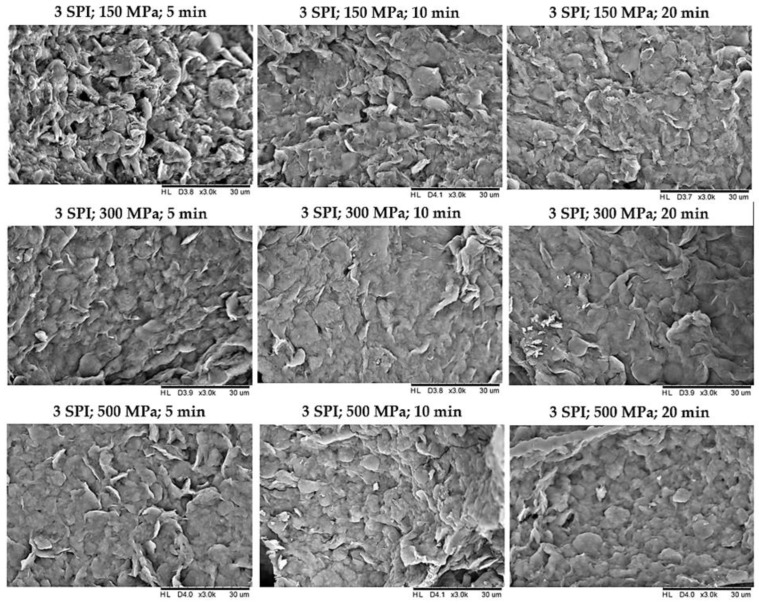
The effect of high hydrostatic pressure induction parameters on the microstructure of inulin–soy protein hydrogels containing 3 g/100 g SPI.

**Figure 15:**
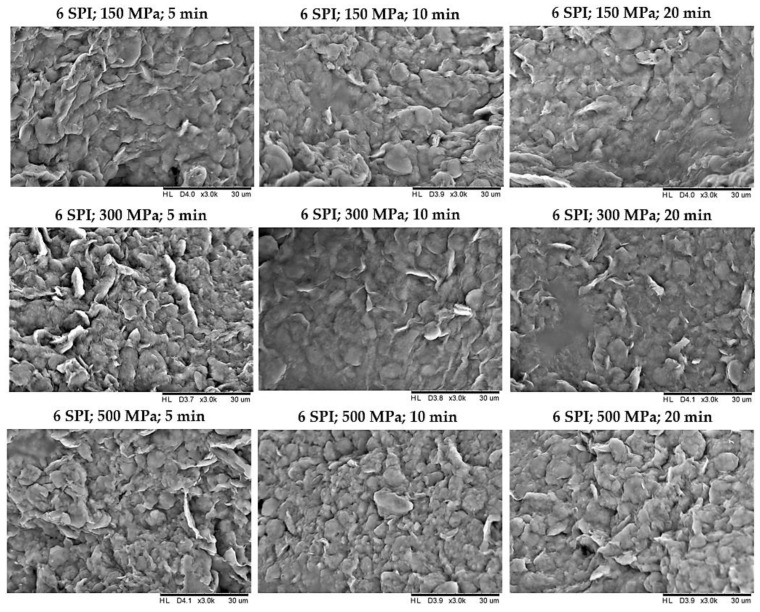
The effect of high hydrostatic pressure induction parameters on the microstructure of inulin–soy protein hydrogels containing 6 g/100 g SPI.

In order to determine how the pressure processing parameters used on inulin–soy protein sols affected the structure of the gels formed, their microstructure was examined using scanning electron microscopy. It was found that modification of the pressure parameters impacted the microstructure of the created gel structures (Figure 14, Figure 15 and Figure S1). The hydrogels obtained with the mildest pressure parameters, i.e., at a pressure of 150 MPa and a treatment time of 5 min, were characterized by the greatest heterogeneity and the most wrinkled structure among the compared gels. Increasing the pressure and extending its treatment time resulted in obtaining gels with a more flattened, compact, and smoother structure, which were also characterized by lower porosity. According to literature data, the structure of inulin gels depends significantly on the pressure used during induction. Comparing the scanning electron microscopy images of hydrogels induced by pressures of 150 and 300 MPa, it was found that the gels induced by a pressure of 150 MPa were loosely packed, without visible melting on the surface of the granules and with a visibly less smooth and less compressed structure [29]. The use of higher pressures, i.e., 500 MPa, resulted in obtaining hydrogels with a smooth surface, the structure of which became uneven and lost its granulation [11]. Analyzing the obtained pictures of the microstructure of inulin–soy protein gels, it was also found that increasing the induction pressure from 150 to 300 MPa had a more significant impact on the microstructure of the gels than an increase from 300 to 500 MPa.

### 2.3. The Effect of High Hydrostatic Pressure Induction Parameters on the Yield Stress and Texture Parameters of the Obtained Hydrogels

#### 2.3.1. Yield Stress

**Figure 16:**
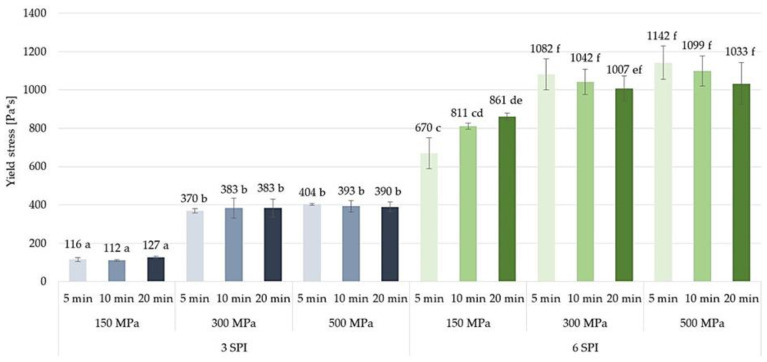
The effect of high hydrostatic pressure induction parameters on the yield stress of inulin–soy protein hydrogels (the average (n = 3) values marked with different letter symbols differ significantly (*p* < 0.05)).

Among the tested hydrogels, the gels obtained from solutions containing 3 g/100 g SPI, subjected to a pressure of 150 MPa, had the lowest yield stress values. Increasing the pressure to which the inulin–soy protein solutions were subjected to 300 MPa resulted in a significant, more than three-fold increase in the yield stress value (Figure 16). Further increasing the pressure to which the sols were subjected to 500 MPa did not result in a further increase in the yield stress of these gels. There was also no significant effect cause by the time of sol pressuring on the yield stress. A significant effect of increasing the pressure to which the inulin–soy protein sols were subjected on the yield stress values was also found in the case of solutions with a higher protein content, i.e., 6 g/100 g SPI. In the case of such solutions, the increase in yield stress as a result of increasing the pressure was, however, smaller than in the case of solutions with 3 g/100 g SPI content. At the same time, as in the case of gels containing 3 g/100 g SPI and also in the case of gels containing a larger amount of this protein, no significant changes were found in the yield stress values between the gels induced by the higher pressure treatments of 300 and 500 MPa. It was also found that in the case of gels induced by a pressure of 150 MPa, extending the time of treatment from 5 to 20 min resulted in a significant increase in the yield stress value of the gels. However, such an effect was not observed when higher pressures were used in gelation induction, i.e., 300 and 500 MPa. Generally, the application of high pressures of the order of 300 and 500 MPa to gels containing proteins or polysaccharides significantly affects their yield stress [11,44]. The pressure compresses the gel network, restricting the movement of the polymer chains that form the gel structure [45,46,47]. This makes it harder for the gel to deform elastically, leading to an increase in the yield point—the stress required for permanent deformation [46,48].

#### 2.3.2. Firmness

**Figure 17:**
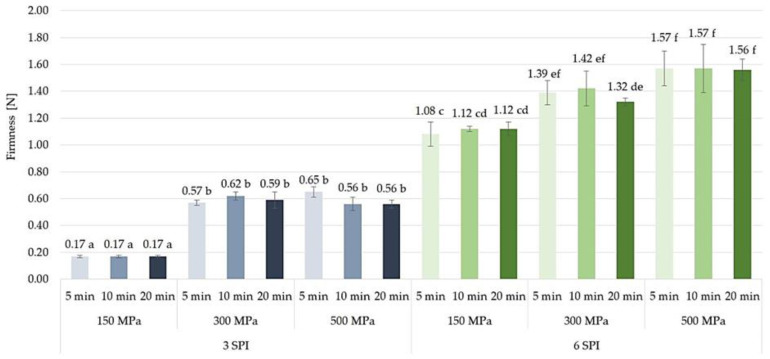
The effect of high hydrostatic pressure induction parameters on the firmness of inulin–soy protein hydrogels (the average (n = 3) values marked with different letter symbols differ significantly (*p* < 0.05)).

It was found that the modification of the firmness of inulin–soy protein hydrogels could be achieved by changing the amount of pressure used for their induction. The softest gels, i.e., those with the lowest firmness values, were obtained by subjecting the sols to a pressure of 150 MPa. In the case of solutions containing 3 g/100 g SPI, subjecting them to higher pressure, i.e., 300 MPa, resulted in obtaining gels with a stronger structure, i.e., firmness values over two and a half times higher (Figure 17). The stronger and more uniform structure of the inulin–soy protein gels obtained as a result of their induction with higher pressure is also indicated by photos of their microstructure (Figure 14). It was also found that for gels containing 3 g/100 g SPI, a further increase in the pressure used for induction, i.e., from 300 to 500 MPa, did not result in a further increase in the firmness of the obtained gels. There was also no significant effect of the high-pressure induction time on the firmness of the obtained inulin–soy protein gels. Similar trends in the influence of high pressure on the firmness of the obtained gels were found in the case of a higher SPI content, i.e., 6 g/100 g. It was found that in most of the analyzed cases, increasing the induction pressure from 150 to 300 MPa allowed inulin–soy protein gels with higher firmness values to be obtained. It was also found that when pressurizing solutions with a higher protein concentration (i.e., 6 g/100 g), the increase in firmness obtained by increasing the induction pressure from 150 to 300 MPa was smaller than in the case of solutions with a lower protein content. As in the case of gels with 3 g/100 g SPI, and also in the case of gels with a higher protein concentration, there was no significant effect of further increasing the gel induction pressure (i.e., from 300 to 500 MPa) on the firmness of the gels when acting for 5 and 10 min. Only in the case of the longest exposure of high pressure to inulin–soy protein sols (i.e., 20 min) was it found that there was a statistically significant increase in the firmness of the gels as a result of increasing the induction pressure from 300 to 500 MPa. According to the literature data, the effect of high pressure on protein–polysaccharide gels may vary depending on the polymers used, the pressure level, and the pressure duration [43,49]. In gels formed by proteins, high pressure can strengthen the protein network. This is because pressure can promote interactions between protein molecules, leading to a stiffer structure and a harder gel [50,51]. However, high pressure can have the opposite effect on polysaccharide gels. For example, by exposing starch gels to high pressures, Liu et al. [52] and Ji et al. [53] found that initially applying pressure lowered the gelatinization temperature (the point at which the starch thickens). In contrast, prolonged exposure could weaken the gel network over time. Similar observations were also made for carrageenan gels [54].

#### 2.3.3. Spreadability

**Figure 18:**
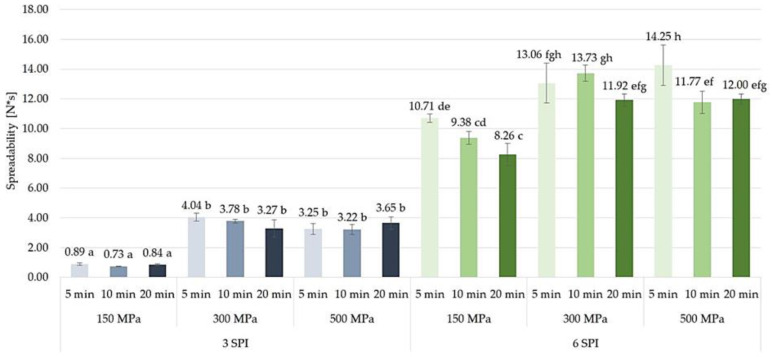
The effect of high hydrostatic pressure induction parameters on the spreadability of inulin–soy protein hydrogels (the average (n = 3) values marked with different letter symbols differ significantly (*p* < 0.05)).

By influencing the structure, the modification of the macromolecular arrangements of protein, the polysaccharide gelation, and other interactions between food ingredients, high pressures also affect the spreadability of gels obtained from proteins and polysaccharides [55,56,57,58]. It was found that the highest spreadability (i.e., the lowest spreadability values, at the level of 0.73–0.89 N*s) was observed in hydrogels containing 3 g/100 g of SPI, subjected to a pressure of 150 MPa. The increase in pressure to which the sols were subjected from 150 to 300 MPa resulted in a 3.89-to-5.18-fold decrease in the spreadability of the formed gels (Figure 18). A further increase in the gelation induction pressure of inulin–protein sols, i.e., from 300 to 500 MPa, did not cause any further changes in the spreadability of the formed hydrogels. It was also found that the time of exposure of the sols to high pressure had no significant effect on the spreadability values of the formed hydrogels. When analyzing inulin–protein hydrogels with a higher SPI content, i.e., 6 g/100 g, it was found that, similarly to gels containing 3 g/100 g SPI, increasing the pressure used to induce gelation of the inulin–protein systems resulted in the formation of gels with lower spreadability. However, in the case of gels containing a higher amount of protein (i.e., 6 g/100 g SPI), it was found that the differences in the spreadability of inulin–protein hydrogels induced at a pressure of 150 and induced at a pressure of 300 MPa were smaller than in the case of gels with a lower protein content (i.e., 3 g/100 g SPI). It was also found that for hydrogels with a higher protein content (i.e., 6 g/100 g SPI) induced by pressures of 150 and 500 MPa, extending the high pressure exposure time from 5 to 20 min resulted in a significant increase in their spreadability. However, such a clear effect was not observed for inulin–protein gels induced by a pressure of 300 MPa.

#### 2.3.4. Adhesiveness

**Figure 19:**
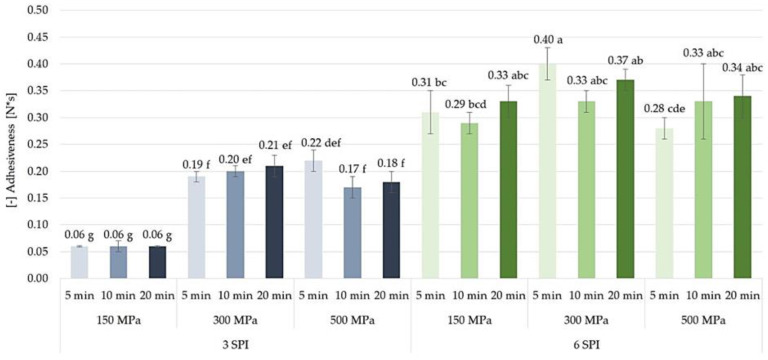
The effect of high hydrostatic pressure induction parameters on the adhesiveness of inulin–soy protein hydrogels (the average (n = 3) values marked with different letter symbols differ significantly (*p* < 0.05)).

It was found that increasing the pressure used to induce the gelation of inulin–protein systems containing 3 g/100 g SPI from 150 to 300 MPa resulted in the formation of gels with several times (i.e., from 3.17 to 3.5 times) greater adhesiveness (Figure 19). However, further increasing the pressure to which the sols were subjected (i.e., from 300 to 500 MPa) did not result in a further increase in the adhesiveness of the gels. In the case of gels with a higher protein content (i.e., 6 g/100 g SPI), it was found that only during the shortest high pressure sol exposure time (i.e., 5 min) did increasing the induction pressure from 150 to 300 MPa result in a significant increase in the adhesiveness of the formed gels. Such an effect was not observed with a longer pressurization time. No significant changes in the adhesiveness of inulin–protein gels containing 6 g/100 g SPI were observed as a result of increasing the pressure used for their induction from 300 to 500 MPa. It was also found that the adhesiveness values of HHP-induced inulin–protein gels were not significantly influenced by the time of high pressure exposure, both in the case of gels with lower (i.e., 3 g/100 g) and higher (i.e., 6 g/100 g) protein content. High-pressure processing assists in gelation, resulting in the improved mechanical properties of gels. The pressure-induced modification of macromolecular arrangements contributes to the enhancement of adhesiveness in gels. Longer holding times were found to lead to more pronounced effects on gel structure, including increased β-sheet content and improved mechanical properties of the gels [44,59,60].

### 2.4. The Effect of High Hydrostatic Pressure Induction Parameters on the Color and the Total Color Difference Parameters of the Obtained Hydrogels

**Figure 20:**
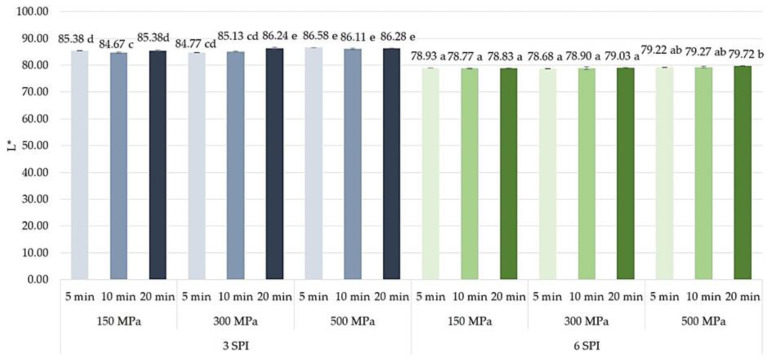
The effect of high hydrostatic pressure induction parameters on the lightness (color L* parameters) of inulin–soy protein hydrogels (the average (n = 3) values marked with different letter symbols differ significantly (*p* < 0.05)).

When analyzing the effects of inulin–protein sol pressuring parameters on the lightness (L*) of the color of the formed gels, it was found that in the case of systems with lower protein content (i.e., 3 g/100 g SPI), increasing the induction pressure from 150 to 500 MPa caused a small, although significant, increase in the lightness of the formed gels (Figure 20). In the case of the longest time of high pressure exposure (i.e., 20 min), such an effect was already observed when the pressure was increased from 150 to 300 MPa. For inulin–protein systems with higher protein content, such a significant increase in the lightness of the formed hydrogels was observed only when the highest pressure (i.e., 500 MPa) was applied for the longest time (i.e., 20 min). The use of milder gelation induction parameters for inulin–protein systems did not result in a significant increase in the color parameter L* of the hydrogels. In most of the analyzed variants of the formed gels, no significant effect of the sol pressurization time on the lightness of the color of the formed gels was found. Only a slight lightening of the gels containing 3 g/100 g SPI, induced by pressures of 150 and 300 MPa, was observed due to the extension of the HHP exposure time from 10 to 20 min. According to the literature, the effect on lightness is more pronounced at higher pressures or longer holding times, especially if this leads to significant changes in the gel network [61]. Higher pressures lead to denser gels with reduced lightness. The exact relationship can vary depending on the specific gel formulation and the concentration of ingredients [44,62].

**Figure 21:**
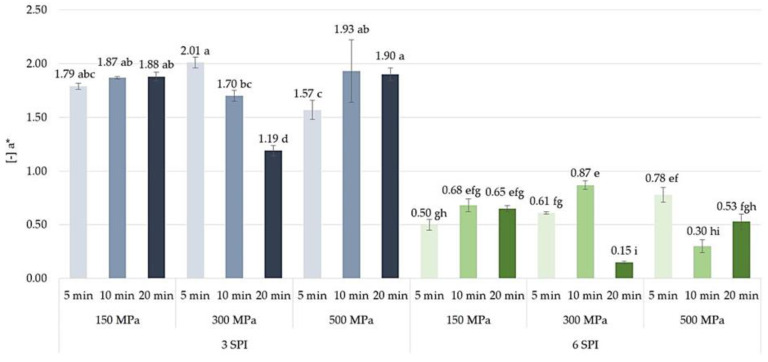
The effect of high hydrostatic pressure induction parameters on the color a* parameters of inulin–soy protein hydrogels (the average (n = 3) values marked with different letter symbols differ significantly (*p* < 0.05)).

**Figure 22:**
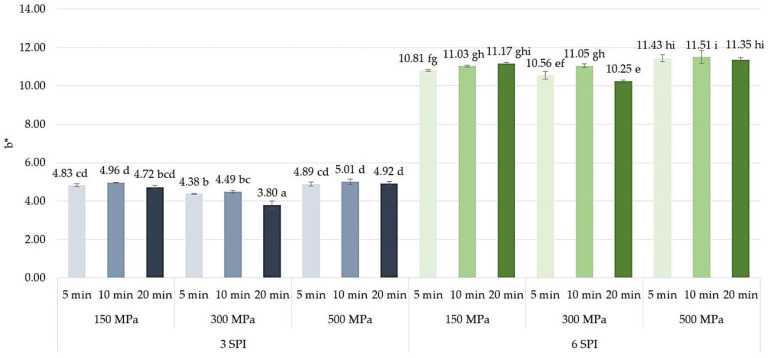
The effect of high hydrostatic pressure induction parameters on the color b* parameters of inulin–soy protein hydrogels (the average (n = 3) values marked with different letter symbols differ significantly (*p* < 0.05)).

When analyzing the effect of using high pressure to induce the gelation of inulin–protein systems on the color of the formed hydrogels, no clear effect of either the amount of pressure applied or the time of its action on the values of the color parameter a* was found (Figure 21). The average values of this color parameter were within a narrow range, i.e., from −2.01 to −1.19 for hydrogels containing 3 g/100 g of SPI and from −0.87 to −0.15 for hydrogels containing 6 g/100 g of SPI. In the case of high-pressure treatment for 5 and 10 min, no significant differences in the values of this color parameter were found between the gels induced by a pressure of 150 MPa and those induced by a pressure of 300 MPa. However, with a longer pressurization time (i.e., 20 min), a significant increase in the value of the color parameter a* was observed. However, increasing the induction pressure from 300 to 500 MPa with an exposure time of 20 min caused the opposite trend, i.e., a decrease in the value of this color parameter. The studies conducted did not show any clear influence of high pressure on inulin–protein sols on the b* color parameter of hydrogels. The values of this color parameter were similar and ranged from 3.80 to 5.01 for gels containing 3 g/100 g of SPI and from 10.25 to 11.51 for gels containing 6 g/100 g of SPI. It was found that in the case of inulin–protein systems containing 3 g/100 g of SPI, an increase in induction pressure from 150 to 300 MPa resulted in a slight decrease in the value of the b* color parameter of the formed gels. In contrast, a further increase in induction pressure, i.e., from 300 to 500 MPa, resulted in a slight increase in the value of this color parameter (Figure 22). The tendency to increase the value of the b* color parameter of the gels as a result of increasing the pressure used to induce the gelation of inulin–protein systems from 300 to 500 MPa was also observed for protein systems with higher protein content (i.e., 6 g/100 g SPI). Analyzing the effect of pressure time on the values of the b* color parameter, it was found that only in the case of hydrogels induced by a pressure of 300 MPa did extending the pressure time from 10 to 20 min result in the formation of gels with a lower share of this color parameter.

In order to comprehensively express the differences in the color of gels induced by high pressures of 150, 300, and 500 MPa, applied for 5, 10, and 20 min, the total color difference coefficient was also calculated. It was found that in most cases, it took values below 2.0 (in the case of 3 g/100 g SPI gels, values from 0.2 to 2.1, and in the case of 6 g/100 g SPI gels, values from 0.2 to 1.4) (Table 1), which means that the differences in the color of the gels induced at different HHP parameters were not visible or were only noticeable by an experienced observer. The effect of HHP on the components of gel color depends to the greatest extent on the components that constitute the gels. Very often, the influence of the parameters of the applied pressure is significant for the color change [28]. However, the available literature also describes studies in which there is no such influence [63].

### 2.5. Principal Component Analysis (PCA)

**Figure 23:**
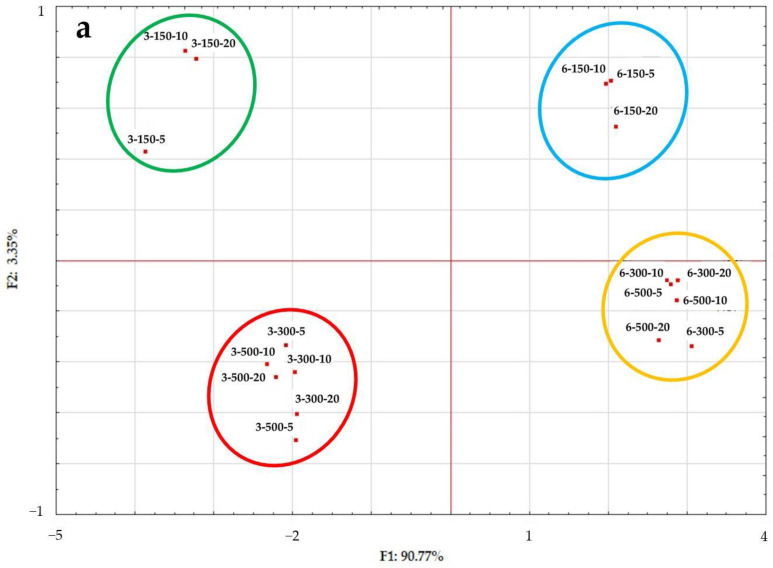

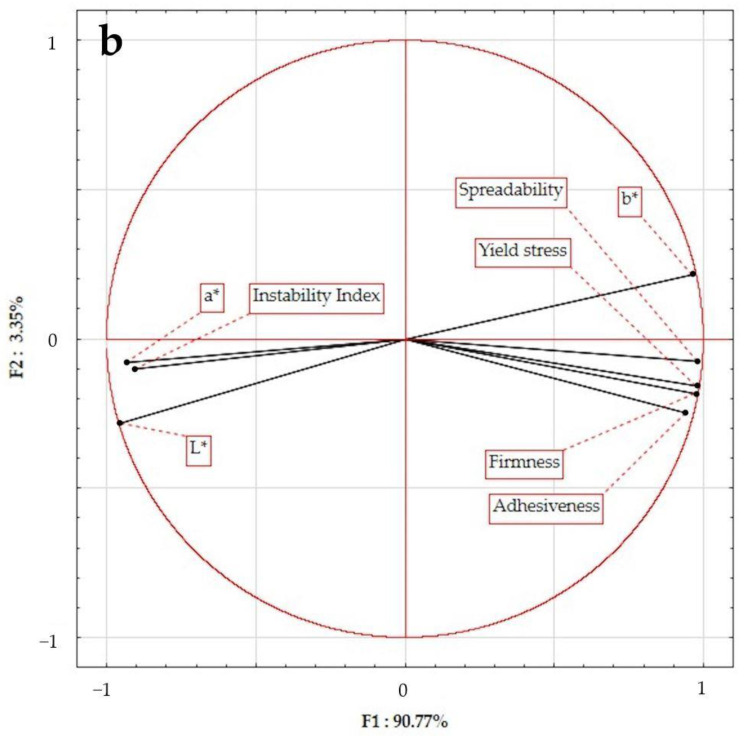
Principal component analysis (PCA): (**a**) Score plot: F1 versus F2 of all samples. (**b**) Score plot: F1 versus F2 of data from determinations used as variables.

In order to determine the most important factors that differentiate samples, the obtained results were subjected to a principal component analysis (PCA). Figure 23 shows the two principal components identified: Component 1 (F1), explaining 90.77% of the variance, and Component 2 (F2), explaining 3.35% of the variance. Component 1 is strongly positively correlated with texture parameters such as firmness (r = 0.97), adhesiveness (r = 0.94), and spreadability (r = 0.98), and also with the yield stress (r = 0.98). In contrast, the instability index (r= −0.91) and the L* (r= −0.95) and a* (r= −0.94) color parameters were negatively impacted by this factor. Based on the hydrogels’ distribution across the space of the principal components, four clusters were distinguished. Based on them, it can be stated that the greatest differences in the properties of the tested inulin–protein hydrogels occurred between samples induced at a pressure of 150 MPa (green and blue clusters) and those induced at higher pressures, i.e., 300 and 500 MPa (red and yellow clusters). These differences were found regardless of the high-pressure treatment time and protein concentration.

In the available literature, additional attention is also paid to the influence of the applied pressure in relation to the protein content in the system. As indicated by the research of Wang et al. [65], at lower (3%) protein concentrations, unfolded globulins remain stable, and hydrophobic group exposure is mainly determined by the pressure level. At higher concentrations (more than 5%), unfolded proteins readily associate to form aggregates. High-pressure treatment at lower pressures partially unfolds proteins, while higher pressures lead to complete unfolding and subsequent re-association into soluble complexes. The emission fluorescence spectra peak intensity of 1 and 3% soy protein isolate (SPI) increases with pressure, indicating the exposure of hydrophobic groups. SPI 5% shows a similar trend up to 400 MPa, but then decreases, suggesting re-association or aggregation. According to the authors, glycinin is more easily denatured by high-pressure treatment than beta-conglycinin, especially at higher protein concentrations. However, high-pressure treatment at 400 MPa almost completely denatures glycinin at various SPI concentrations [66].

## 3. Conclusions

The use of HHP, even at the lowest tested parameters (i.e., 150 MPa, 5 min), allowed the effective induction of the formation of inulin–soy protein hydrogel structures. However, the gels obtained with such parameters had a delicate structure. The use of a higher induction pressure, i.e., 300 MPa, allowed us to obtain gels with a more flattened, compact microstructure, a stronger structure, and lower spreadability, especially in systems with a lower soy protein content (3 SPI). A further increase in the induction pressure (up to 500 MPa), as well as, in most cases, an extension of its exposure time (up to 20 min), did not cause any further significant changes in the microstructure and rheological characteristics of the formed hydrogels. The obtained results indicate that HHP, using the parameters of 300 MPa and 5 min, can be used as an effective method for strengthening the structure of inulin–soy protein hydrogels. The use of higher pressure and a longer gelation induction time is not necessary, and in the case of hydrogels with a higher soy protein content (6 g/100 g SPI), it is even inadvisable, as it may lead to a deterioration of their stability.

The results obtained during our studies indicate the possibility of using high-pressure processing of solutions containing inulin and soy protein to create hydrogels without needing high-temperature processing. Potentially, such gels can be used in the creation of functional food, which can be a matrix for introducing nutritionally valuable but thermolabile ingredients into food. An additional advantage of such hydrogels may be the controlled release of bioactive ingredients in the distal parts of the digestive system. Future research will focus on determining the changes that will be caused by the introduction of a bioactive ingredient into the hydrogel matrix.

## 4. Materials and Methods

### 4.1. Materials and Preparation of Hydrogels

In order to prepare the hydrogels for the analysis, inulin (INU; Inulin HPX Beneo, ORAFTI, molecular weight 5600–6300 g/mol) was used in concentrations of 20 g/100 g, and soy protein isolate (SPI; MyProtein, molecular weight 194.23 g/mol) was used in concentrations of 3 or 6 g/100 g. The particular stages of gel production were:

- Preparation of an aqueous solution of inulin by mixing it with water;
- Addition of the appropriate protein concentration, and preparing the suspension;
- Pouring the samples into plastic bottles used in the high-pressure chamber for pressurization;
- Subjecting samples to high-pressure treatments (apparatus U 5000/120 Unipress, Warsaw, Poland), using pressures of 150, 300, or 500 MPa and time treatments of 5, 10, or 20 min.

In order to form a gel structure, the gel samples were left, after pressurization, at refrigeration temperature (8 ± 1 °C) for 24 h. Before analysis, the samples were conditioned to a temperature of 20 ± 1 °C. The exception was the measurement of microrheological properties, which was performed immediately after the HHP induction. The study was performed in triplicate (n = 3).

### 4.2. Methods

To describe the degree of gelation of the tested samples, the volumetric gel index (VGI) was analyzed. VGI describes the degree of gel formation as the gel volume over the total volume of the sample. If there was no gel formed, the sample’s VGI was 0; the value of this index, between 0 and 100%, corresponded to the percentage of gel formed [26].

Microrheology was analyzed by multi-speckle diffusing-wave spectroscopy (MS-DWS), where the detector captures the interfering backscattered waves. For this purpose, the Rheolaser Master device (Formulation, Toulouse, France) was used. The following parameters were selected for analysis: wavelength 650 nm, measuring time 23 h, and temperature 22 ± 1 °C. The measurement results were recorded and visualized using the Rheotest software RheoSoft Master_v1.4.0.10. During the analysis, the following measurements were taken—Mean Square Displacement (MSD) (nm^2^), elasticity index (EI) (nm^−2^), Solid–Liquid Balance (SLB), Macroscopic Viscosity Index (MVI) (nm^−2·^·s), and fluidity index (Hz) [66].

The stability of the tested hydrogels was analyzed using a photo-optical centrifuge, the LUMiSizer 6120-75 (L.U.M. GmbH, Berlin, Germany). The parameters used during the study were as follows: wavelength: 870 nm; volume: 1,8 mL of the dispersion; light factor: 1; 1500 rpm; experiment time: 15 h 10 min; interval time: 210 s; temperature: 20 ± 1 °C.

Microstructure was analyzed using an electron scanning microscope TM3000 (Hitachi High-Technologies Corporation, Tokyo, Japan) and digital image recording. The hydrogels were previously freeze-dried and then placed on the double sticky film on the microscope table. The next step was to cover with gold. The images were taken by a built-in light–color optical navigation camera. Specimens were observed at pressures of 100 kPa under an accelerating voltage of 10 kV.

Yield stress [Pa*s] was measured using a rheometer (DV3T, Brookfield, Middleboro, MA, USA) equipped with a vane spindle V74 with a torque range HA. The parameters of the measurements used in the experiments were as follows: the rotation speed was adjusted to rise by 0.10 RMP continuously until the gel structure reached its flow limit.

In order to measure the textural properties of the tested hydrogel samples, texture measurements were performed using a texture analyzer (TA.XT Plus, Stable Micro Mixtures, Surrey, UK). The measuring device was equipped with a 0.5 cm diameter cylindrical flat probe (P/0.5R) to measure the hydrogel textural parameters such as firmness (N) and adhesiveness (N*s). The parameters used during the measurement are: speed 1.0 mm/s, penetration depth—8 mm. In turn, the spreadability of the hydrogels (N*s)was measured using the TTC Spreadability Rig and parameter that was set was speed at level 3.0 mm/s.

The color parameters of the obtained hydrogels were determined using a Minolta CR-400 colorimeter (Minolta, Tokyo, Japan). The parameters used during the measurement were the light source D65, and a measuring head hole of 8 mm. The color components were measured in the CIEL*a*b* system. In order to comprehensively determine the differences in the color of the formed hydrogels, the total color difference parameter was calculated as ΔE. The total color difference was calculated as follows [64]:

$$∆E=\sqrt{{\left(L_{1}^{*}-L_{2}^{*}\right)}^{2}{+(a_{1}^{*}-a_{2}^{*})}^{2}{+(b_{1}^{*}-b_{2}^{*})}^{2}}$$

where L_1_*, a_1_*, b_1_* and L_2_*, a_2_*, b_2_* refer to the color parameters of the compared hydrogels.

The following values of the ΔE ranges were adopted for the interpretation of the obtained results [64]: 0 < ΔE < 1—meaning that a difference is not noticeable for the observer; 1 < ΔE < 2—meaning that only experienced observers can notice the difference; 2 < ΔE < 3.5—meaning that inexperienced observers will also notice the difference; 3.5 < ΔE < 5—meaning that a clear color difference is noticed; and (5 < ΔE)—meaning that two different colors are noticed.

Statistical Analysis—the data (n = 3) were statistically evaluated using the Statistica 13.3 (TIBCO Software Inc. Palo Alto, CA, USA) software. A one-way analysis of variance was performed to assess the significance of differences in the average values of measured parameters of the inulin–soy protein hydrogels. Tukey’s test at a significance level α = 0.05 revealed significant differences between hydrogels obtained in different induction parameters. Additionally, the results were also assessed using principal component analysis (PCA).

## Figures and Tables

**Table 1:** The effect of high hydrostatic pressure induction parameters on the total color differences of inulin–soy protein hydrogels.

Parameters			3 SPI									6 SPI							
			150 MPa			300 MPa			500 MPa			150 MPa			300 MPa			500 MPa	
			5 min	10 min	20 min	5 min	10 min	20 min	5 min	10 min	20 min	5 min	10 min	20 min	5 min	10 min	20 min	5 min	10 min
6 SPI	500 MPa	20 min	8.7	8.2	8.8	8.7	8.8	10.0	9.5	9.1	9.3	1.0	1.0	0.9	1.3	0.9	1.4	0.6	0.5
		10 min	9.2	8.6	9.3	9.2	9.3	10.4	10.0	9.6	9.8	0.8	0.8	0.7	1.2	0.8	1.3	0.5	
		5 min	9.1	8.5	9.2	9.1	9.2	10.4	9.9	9.5	9.7	0.7	0.6	0.5	1.0	0.5	1.4		
	300 MPa	20 min	8.5	7.9	8.6	8.4	8.5	9.7	9.4	9.0	9.2	0.7	1.0	1.1	0.7	1.1			
		10 min	9.0	8.4	9.1	9.0	9.1	10.3	9.9	9.5	9.7	0.4	0.2	0.3	0.6				
		5 min	8.9	8.3	9.0	8.8	8.9	10.2	9.8	9.4	9.6	0.4	0.5	0.6					
	150 MPa	20 min	9.2	8.6	9.3	9.1	9.3	10.5	10.0	9.6	9.8	0.4	0.2						
		10 min	9.1	8.5	9.2	9.1	9.2	10.4	10.0	9.6	9.8	0.3							
		5 min	8.9	8.3	9.0	8.8	8.9	10.2	9.7	9.3	9.5								
3 SPI	500 MPa	20 min	0.9	1.6	0.9	1.6	1.2	1.3	0.5	0.2									
		10 min	0.8	1.5	0.8	1.5	1.1	1.4	0.6										
		5 min	1.2	1.9	1.3	1.9	1.5	1.2											
	300 MPa	20 min	1.5	2.1	1.4	1.8	1.4												
		10 min	0.4	0.7	0.4	0.5													
		5 min	0.8	0.6	0.7														
	150 MPa	20 min	0.2	0.8															
		10 min	0.7																

Note: The color difference between the samples can be estimated based on the ΔE values [64]: if 0 < ΔE < 1, the color is determined as not noticeable for the observer; if 1 < ΔE < 2, only experienced observers can notice the difference in colors; if 2 < ΔE < 3.5, inexperienced observers can also notice the difference in colors; if 3.5 < ΔE < 5, a clear color difference in colors is noticed; and if 5 < ΔE, the observer notices two different colors.

## Data Availability

The data used to support the findings of this study can be made available by the corresponding author upon request.

## Supplementary Materials

The following supporting information can be downloaded at: https://www.mdpi.com/article/10.3390/gels10090570/s1, Figure S1: Physical map of the inulin-soy protein hydrogels obtained by high hydrostatic pressure induction with different parameters.
