# Supplementary figures and images for: The Effect of High Hydrostatic Pressure (HHP) Induction Parameters on the Formation and Properties of Inulin–Soy Protein Hydrogels
Source: Gels. 2024 Aug 31;10(9):570. doi: 10.3390/gels10090570 (PMC11430855; doi:10.3390/gels10090570)

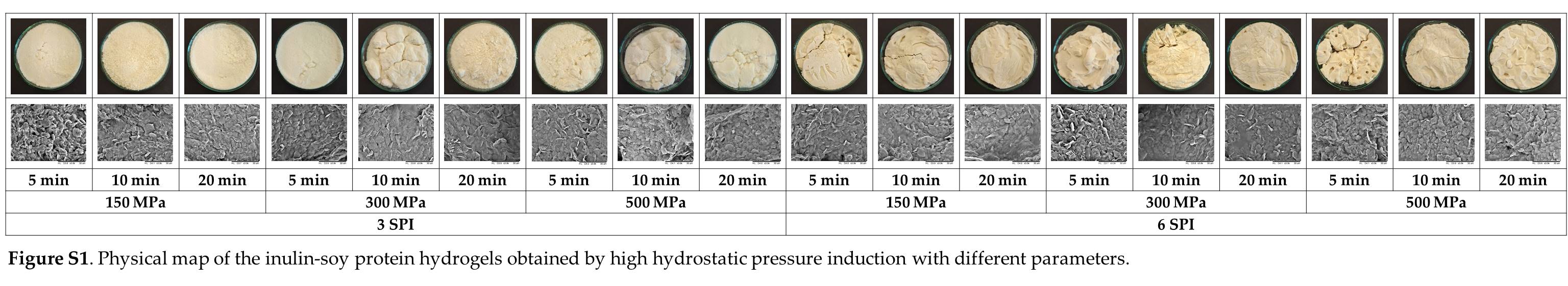

Supplement: Supplementary file 1 [file gels-10-00570-s001.zip › gels-3142313-supplementary.jpg]
